# Dynamic Covalent Hydrogels: Strong yet Dynamic

**DOI:** 10.3390/gels8090577

**Published:** 2022-09-10

**Authors:** Yueying Han, Yi Cao, Hai Lei

**Affiliations:** 1 Collaborative Innovation Center of Advanced Microstructures, National Laboratory of Solid State Microstructure, Department of Physics, Nanjing University, Nanjing 210093, China; 2 Chemistry and Biomedicine Innovation Center (ChemBIC), Nanjing University, Nanjing 210023, China; 3 Jinan Microecological Biomedicine Shandong Laboratory, Jinan 250021, China

**Keywords:** dynamic covalent chemistry, hydrogel, single molecule force spectroscopy, liquid-like properties

## Abstract

Hydrogels are crosslinked polymer networks with time-dependent mechanical response. The overall mechanical properties are correlated with the dynamics of the crosslinks. Generally, hydrogels crosslinked by permanent chemical crosslinks are strong but static, while hydrogels crosslinked by physical interactions are weak but dynamic. It is highly desirable to create synthetic hydrogels that possess strong mechanical stability yet remain dynamic for various applications, such as drug delivery cargos, tissue engineering scaffolds, and shape-memory materials. Recently, with the introduction of dynamic covalent chemistry, the seemingly conflicting mechanical properties, i.e., stability and dynamics, have been successfully combined in the same hydrogels. Dynamic covalent bonds are mechanically stable yet still capable of exchanging, dissociating, or switching in response to external stimuli, empowering the hydrogels with self-healing properties, injectability and suitability for postprocessing and additive manufacturing. Here in this review, we first summarize the common dynamic covalent bonds used in hydrogel networks based on various chemical reaction mechanisms and the mechanical strength of these bonds at the single molecule level. Next, we discuss how dynamic covalent chemistry makes hydrogel materials more dynamic from the materials perspective. Furthermore, we highlight the challenges and future perspectives of dynamic covalent hydrogels.

## 1. Introduction

Hydrogels are soft materials that have the ability to hold large volumes of water, combining the properties of both solid and liquid. Owing to their inherent outstanding biocompatibility/biodegradability, similar mechanical properties to native tissues and organs, and environmental responsiveness [1,2], hydrogels have been largely explored for numerous biomedical and tissue regeneration applications, including drug delivery, biosensors, and synthetic extracellular matrix (ECM) for tissue engineering and regenerative medicine, as well as contact lenses and soft robotics [3,4,5,6]. The applications of hydrogels mainly depend on their mechanical properties, such as modulus, stiffness, toughness, viscoelasticity, porosity, self-healing, adaptivity, and so on. These properties are determined by the hydrogel network design, especially by the crosslinks in the network [5]. Typically, both synthetic and natural polymers have been used to prepare hydrogels, and they are crosslinked physically or chemically. Synthetic hydrogels use various chemistries to crosslink synthetic polymer molecules, resulting in hydrogels with relatively high mechanical strength. The most widely used crosslinking reactions include free radical polymerization, and orthogonal coupling reactions such as thiol–maleimide reactions, and alkyne–azide reactions [7,8,9,10]. In addition, many biomolecules, including proteins, DNAs, and RNAs, have been used as building blocks for synthetic hydrogels through chemical crosslinking [11,12]. These covalently crosslinked hydrogels are called chemical hydrogels, and cannot be dissolved in solvents unless covalent bonds are cleaved or the polymers are degraded. Although permanently crosslinked covalent hydrogels possess relatively high mechanical strength, they are difficult to be remolded once formed. Therefore, they are not suitable for many applications, especially for cell culture and tissue engineering. On the other hand, physical hydrogel networks are crosslinked by molecular entanglements and non-covalent chemistries, such as ionic interactions, hydrogen-bonding, hydrophobic interactions, π–π stacking, and host–guest interactions [13,14,15,16,17,18]. All these interactions are reversible, and the mechanical properties can be altered by changes in pH, temperature, light, stress, and the addition of specific chemical or mechanical stimuli. However, physical hydrogels usually have poorer mechanical properties than that of chemical hydrogels, as the physical interactions are weaker than the covalent bonds. Therefore, hydrogels combining the mechanical properties of chemical hydrogels and physical hydrogels have attracted more attention in recent studies. The most conventional method to achieve this goal is to use dynamic covalent chemistry (DCC) in hydrogel networks [19,20]. 

**Figure 1:**
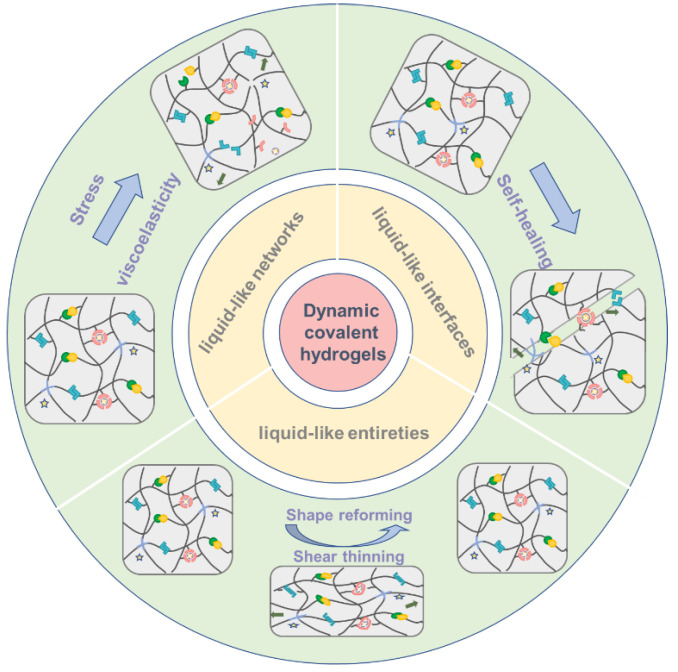
Dynamic covalent chemistry endows hydrogels with more liquid-like properties.

Dynamic covalent bonds can break, recombine, and exchange reversibly under certain stimuli [21,22]. This reversible process is similar to physical interactions, and can also help hydrogel materials dissipate energy [23,24]. In addition, the bond energy of DCC is higher than that of physical interactions, and the use of DCC in hydrogel network can increase the micro dynamics of the network while maintaining its macro stability. Introducing DCC into a hydrogel network will make it more liquid-like from micro (network and topological structure) to macro (interface and entirety) scale, and acquire unique properties. By doing so, the dynamic covalent hydrogels (DCHs) not only maintain relative mechanical strength, but also respond to external biochemical/physical stimuli, which broadens their applications in biomedicines and biotechnologies. The DCC makes the hydrogels with properties including self-healing, shape memory and controllable stiffness, and an excellent review of this is provided elsewhere [25,26,27]. In this review, we first introduce the common dynamic covalent bonds based on various mechanisms and their mechanical properties at the single molecule level. Then, we focus on how dynamic covalent bonds make the material “liquid-like” at three levels: network, interface and entirety, and also illustrate the applications of dynamic covalent hydrogels (Figure 1). Finally, we will put forward new expectations for dynamic covalent bond hydrogel materials.

## 2. Dynamic Covalent Chemistry

**Figure 2:**
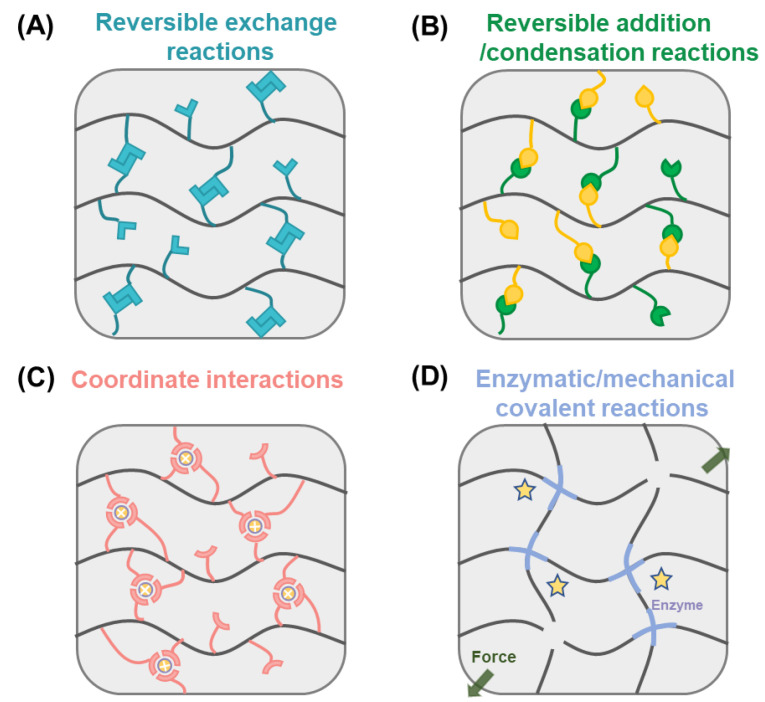
Schematics of dynamic covalent chemistry for hydrogels. (**A**) reversible exchange reactions, including disulfide, diselenide, and thioester; (**B**) reversible addition/condensation reactions, such as imine, hydrozone, oxime, and boronic ester; (**C**) coordinate interactions and (**D**) enzymatic/mechanical covalent reactions.

Dynamic covalent chemistry is different from permanent bonds because of its dynamic instability and unique intrinsic characteristics. Generally, DCC can be divided into four groups according to chemical mechanisms, as shown in Figure 2, including reversible exchange reactions, reversible addition/condensation reactions, coordinate interactions and enzymatic/mechanical covalent reactions. In addition, the mechanical properties of dynamic covalent bonds are various. When introduced in polymers, such linkage types endow the materials with unique bond features and can also give rise to important emerging properties.

### 2.1. Reversible Exchange

**Figure 3:**
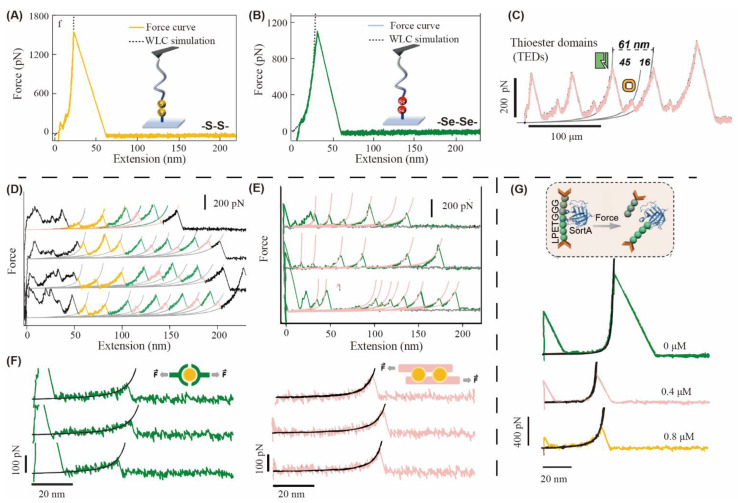
Mechanical measurements of different dynamic covalent bonds using atomic force micro-scope-based single molecule force spectroscopy (AFM-SMFS). Typical force–extension curves of the rupture of (**A**) disulfide bond and (**B**) diselenide bond [32]. Copyright © Editorial Office of Acta Polymerica Sinica; (**C**) schematic diagram of structure and force–extension recordings of the Poly-CnaBD595aTED (D595A (Δiso) construct [38]. Copyright © 2017 by The American Society for Biochemistry and Molecular Biology, Inc.; (**D**) the typical force extension curve from the mechanical unfolding of Fe_2_S_4_ [39]. Copyright © 2017, American Chemical Society (**E**) Study of single-molecule mechanics of catechol-Fe^3+^ complexes by atomic force microscopy [78]. Copyright © 2017, American Chemical Society; (**F**) typical force–extension curves of the rupture of PH_3_-Zn^2+^(green line), and PH_6_-Zn^2+^ (pink line) complexes [40]; (**G**) schematic for the single-molecule force spectroscopy of SrtA-induced cleavage of the LPETGGG peptide under force (top) and typical force-retract curves for the rupture of the LPETGGG peptide in different concentrations of enzyme(bottom) [41]. Copyright © 2022 Wiley-VCH GmbH.

The disulfide bond (S-S) derived from the coupling of thiol groups is one of the most common dynamic covalent bonds through reversible exchange mechanism. The disulfide bond can be formed through an oxidation reaction in the presence of oxidoreductase catalysis and broken via a reduction reaction with a reducing agent such as dithiothreitol (DTT) [28,29]. In the case of proteins, the disulfide bond is always formed between two cysteines. The disulfide bonds play important roles during the protein folding and regulate protein structures and functions in life processes [30,31]. As a covalent bond, the mechanical strength of disulfide bond is strong. Xu and coworkers used the atomic force microscopy (AFM)-based single molecule force spectroscopy (SMFS) to directly observe the rupture of disulfide bond between modified-PEG. The force to break the disulfide bond is nearly 1.4 nN (Figure 3A) [32]. In the contrast, we have measured that the mechanical force of disulfide bond is only about 200 pN in the presence of tris(2-carboxyethyl) phosphine (TCEP) [33]. The mechanical properties of disulfide bond make it an excellent candidate for the dynamic crosslinking of hydrogel networks. However, due to the lack of oxidoreductase catalytic system, the thiol-disulfide bond equilibrium time is long in vitro, the reaction constant is 10^−5^ times that in vivo [34]. When disulfide bonds are introduced into hydrogel networks, oxidants, such as hydrogen peroxide, are usually added to reduce reaction time. Nevertheless, oxidants are usually highly toxic [35]. Recently, we have reported that the gelation time of disulfide hydrogels catalyzed by diselenide compound is shorter than that of air oxidation, and the hydrogels have good biocompatibility for cell embedding and release [36,37].

The diselenide bond (Se-Se) is also a dynamic covalent bond which is more dynamic than a disulfide bond. In terms of bond energy, the bond energy of diselenide bond (172 kJ mol^−1^) is lower than that of disulfide bond (240 kJ mol^−1^) [42]. In addition, the mechanical strength of diselenide bond is approximately 1.1 nN (Figure 3B), which is lower than that of S-S [32]. Therefore, diselenide bonds are more sensitive than those of disulfide bonds. In addition, diselenide bonds can undergo dynamic fracture, exchange and recombination under visible light irradiation (~400 nm), while disulfide bonds require higher energy UV irradiation [43,44]. Diselenide-containing polymers are designed for use in nanodrug delivery systems, and can not only function as drug carriers, but also show good anticancer efficiency and immunomodulatory properties after a quick and sensitive response to oxidative stimuli [45,46,47]. Furthermore, the diselenide bond crosslinked networks exhibit many characteristics, such as self-healing, shape memory, photoplasticity, and photocontrolled information storage [48]. Notably, as a kind of weak bond, diselenide bond is not only responsive to visible light and temperature, but also can break under osmotic pressure and sonication [49,50]. Moreover, the Te atom of the same oxygen group element has a larger radius and higher activity. The Se-Te bond energy is only 167 kJ mol^−1^, and dynamic exchange can occur at wavelengths > 600 nm [51]. In addition to photoresponse, Se-N is also a dynamic covalent bond with ultrasonic response [52,53]. These dynamic bonds can be the new crosslinks for dynamic hydrogel synthesis and enable remote stimulation and regulation of mechanical properties.

In addition, thioester is another dynamic covalent bond that is called precursors to life due to its important roles in life processes. Thioesters are involved in the synthesis of varietal esters in vivo, as well as in the synthesis of other cellular components, including peptides, fatty acids, sterols, and others [54]. In particular, thioesters play the role of ATP in supporting energy production during initial ATP deficiency. There is asymmetric dynamic exchange between mercaptan and thioester. Echelman and coworkers found that the force of thioester domains (TEDs) opening construct is about 100 pN (Figure 3C) [38]. When pH value of solution is higher than thiol pKa, a thiolate anion reacts with a thioester to form new thiolate and thioester products [55]. Hupe and Jencks have proved that the conjugate acid of the incoming thiol and the leaving thiolate anion can determine the sulfur ester exchange rate [56]. The selection of different types of mercaptan allows such dynamic exchange reactions to take place over a wide range of pH values, suitable for both biological and abiotic applications. In recent years, thiol–thioester exchange, or transthioesterification has been introduced into hydrogel networks to increase the dynamic viscoelasticity of networks. Such hydrogels have been well applied and developed for cell packaging in vitro [57,58]. In addition, wound sealing hydrogels utilize thioester exchange to achieve controlled wound closure and degradation [59].

### 2.2. Reversible Addition/Condensation

The Diels–Alder cycloaddition reaction is a cycloaddition reaction, usually involving an electron-rich diene and an electron-poor dienophile [60]. Concentration, pH, temperature, and other environmental factors all affect the reaction rate constants. In addition, Diels–Alder reaction was more suitable at higher temperatures, so the conversion temperature of the thermoreversible polymer network prepared by Wuld and coworkers was up to 120 °C [61]. After modification of the reactants, Diels–Alder hydrogels have been prepared under physiological conditions and maintained dynamic properties. Recent literature has reported that Diels–Alder adducts undergo mechanically induced cycloreversion when loading a mechanical force [62,63]. Through rational computational design, “mechanoresistant” Diels–Alder adducts were identified by constrained geometries simulating external force models and employed to enhance failure strength of crosslinked hydrogels [64].

Schiff base reactions (or more broadly imine reactions) are condensation reactions consisting of the uronic and electrophilic amine, hydrazide, or hydrazine groups, and are the well-known dynamic covalent bonds [65,66,67]. Schiff base is formed by amines (R-NH_2_) and electrophilic carbonyl compounds (R-CHO-R), which have been used to create adaptable materials. Dynamic uncoupling and recoupling of the reversible linkages in imine hydrogels can impart self-healing capability without additional stimuli. Meanwhile, the Schiff base is pH-responsive according to its chemical structure, which can respond to physiological stimuli [68,69]. Then, the reaction kinetics can be adjusted by changing the nucleophile, thus affecting the stability of the network [70]. For example, when hydrazine (R-NH-NH_2_) or hydrazide (R-CH(O)-NH-NH_2_) are used as electrophilic reagents, the resulting hydrazone bond (R-NH-N = C-R) is more stable than imine, and its reversibility adapts to longer time scale [71]. Similarly, alkoxy amine (R-O-NH_2_) and carbonyl group (R-CHO-R) react to form oxime, which is considered to be the most stable and least dynamic imines in cell packaging and tissue engineering [72,73].

Boronic esters or boronates undergo the transesterification reaction between boronic acids and diols [74]. The high rates of the transesterification reaction of borate enable it as a good candidate to construct dynamic materials. In addition, the ability of responding to a variety of external stimuli increases its dynamic property, such as pH, temperature, and other chemical reagents. For example, with an optimal pH range from 7 to 9, boronates can be used to form stable hydrogel. Beyond the pH range, the stability of hydrogel will be significantly reduced and the moduli and relaxation will be greatly changed [75]. In addition, borate esters are sensitive to oxides and can be developed as sensors to detect reactive oxygen and reactive nitrogen in living systems [76]. Therefore, hydrogels containing borates release their internal fluorophores when stimulated by hydrogen peroxide [77]. In contrast, boronic esters and other diol-containing molecules undergo metathesis reaction. Due to the strong combination of borate esters and diol [78], borate esters can be used as cell markers for carbohydrate recognition; moreover, hydrogels containing borate esters can be used for drug delivery in sugar (rich in diol) sensitive system [79,80]. However, as an in vitro cell culture platform, a free-glucose culture environment is needed; otherwise, the gel stability is challenged.

### 2.3. Coordinate Interaction

Coordinate (or dative) bonds are special dynamic covalent bonds, and are a type of covalent bond where both electrons are from the same atom. Typically, it occurs in molecules where metallic ions are bonded to ligands. By changing metal ion or ligands, species dynamics can be tuned. The mechanical strength of many coordination bonds intensities was measured by AFM-based SMFS, which were approximately in the range of the ~10^2^ pN scale [81,82,83]. By using the ferredoxin which includes a Fe_2_S_4_ cluster as model, we have found that the average of mechanical rupture force of Fe-S bond is ~240 pN (Figure 3D) [39]. In addition, we directly quantify the mechanical properties of individual catechol-Fe^3+^ complexes, which display different mechanical strength. The rupture force of the bis-catechol-Fe^3+^ complex is up to 200 pN while the triscatechol-Fe^3+^ complex ruptured at ~100 pN (Figure 3E) [84]. Metal–ligand coordination bonds have very important applications in biological materials. Although metal ions (such as iron, copper, zinc, and calcium) are found in tiny amounts in organisms, play key roles in regulating or maintaining cellular behavior and organism balance. Introducing Ca^2+^, Fe^3+^, Cu^2+^, and Zn^2+^ coordination interactions into hydrogel networks will enhance mechanical strength and tensile properties [85,86]. In our recent work, we have designed a histidine-rich decapeptide containing two tandem zinc binding motifs [40]. The typical force–extension curves for the rupture of PH_3_-Zn^2+^ (green) and PH_6_-Zn^2+^ (pink) complexes are shown in Figure 3F. The rupture forces were 90 pN and 140 pN, respectively. The hydrogel formed with PH_6_-Zn^2+^ complexes combined high strength, ultra-toughness, and fast recovery, making it a potential candidate for cartilage regeneration. Moreover, coordination bonds not only enhance mechanical stability but also allow the material to be sensitive to a range of stimuli, offering more fine-grained control over its properties [87]. These flexible super toughening/super strengthening materials can be used in wide application. For example, Yang and coworkers reported gradient bimetallic ion-based hydrogels via the one-step coordinative crosslinking of sulfhydryl groups with copper and zinc ions, as a microstructure reconstruction for the in-situ tendon-to-bone insertion, which had good mechanical strength, continuous slow-release properties, excellent biocompatibility, and antibacterial properties [88]. Although the binding ability of coordination bond is weaker than that of other dynamic covalent bonds described above, the stability and mechanical properties of hydrogel materials can be improved through reasonable design.

### 2.4. Enzymatic/Mechanical Dynamic Covalent Reactions

In addition, there are other special mechanisms for dynamic covalent chemistry, such as enzymatic/mechanical covalent reactions. The original irreversible chemical bond can be dynamically broken and reformed in the presence of enzyme or under mechanical force. As biocatalyst, enzymes reduce the reaction energy barrier through various mechanisms, avoid the trap of reaction kinetics, and change permanent bonds to dynamic bonds. For example, the sortase A can catalyze the isopeptide bond formation between peptide LPTXG and GGG. On the contrary, this kind of isopeptide would dissociate with high concentration of sortase A. Recently, we have used the AFM-based SMFS to quantify the mechanical strength of this reversible bond. As shown in Figure 3G, along with the increase of enzyme (sortase A) concentration, the rupture force of peptide decreased from 1100 pN to 270 pN. Next, we use this dynamic covalent bond to design a dynamic hydrogel whose properties can be precisely controlled at the molecular level [41]. Enzymes play a very important role in the healing of living organisms, such as regulating DNA mitosis, epidermal cell proliferation and migration [89,90,91]. Inspired by this, self-healing hydrogels can be prepared by rational design with enzymatic reactions. Enzymatic reactions can balance competing reactions and improve internal self-healing ability. Monoamine oxidase B (MAO B), catalase (CAT), plasma amine oxidase (PAO), and urease have been reported to regulate the self-healing properties of dynamic covalent hydrogels [92,93,94,95]. In particular, another advantage of hydrogels for enzymatic reactions is that hydrogels provide a protective environment for the enzyme, which avoids enzyme denaturation and inactivation in inferior conditions [96,97]. Forces can also be involved in dynamic chemism regulation. Yao et al. measured the force response ring-opening of spirothiopyran (STP) based on SMFS. Under the loading force of 380 pN force, STP opens the ring and closes the ring again after the force is removed, which can be used in self-healing materials [98].

### 2.5. Properties of Dynamic Covalent Polymers

Reversible bond formation is the key feature of DCC [99]. In a system with dynamic covalent bonds, the forward (bonding) reaction and the reverse (dissociation) reaction take place simultaneously, resulting in the most stable thermodynamic state [100]. When the system is stimulated by the corresponding physical, chemical, or biological stimuli, the dynamic covalent bonds rupture and break the original equilibrium. In principle, dynamic bonds reform and restore the polymer in its original physical form and functionality after removing the stimulus. Therefore, in material design, building blocks need to be considered and selected comprehensively. In this way, the polymerization reaction of materials is dominant, which can guarantee to dynamically regulate the properties of materials, including self-healing and recyclability [101,102]. Unlike the healing mechanisms of traditional material, dynamic polymers are able to spontaneously recover from damage to their original form without external agents in principle. Common covalent polymers are unusable, while limited by the weak interactions of non-covalent materials, although the structure and mechanical properties can be recycled, it is hard to form a strong mechanical structure. Dynamic covalent materials contain both recyclability and robust characters [103].

Dynamic covalent polymer network is adaptive, which is related to the equilibria of DCC, and the network structure changes after the surrounding environmental factors changing [104]. Dynamic covalent bonds can respond to physical stimulus such as pH, sound, light, electricity, and heat, as well as to chemical stimulus such as oxidants, reductants and sugars, etc. The application of bifunctional materials is suitable for gene delivery and biorecognition [105,106]. For example, disulfide bonds are responsive to reducing agents, and hydrogels constructed with disulfide bonds as crosslinkers have a good application in tumor drug delivery [107,108]. In the tumor microenvironment, glutathione (GSH) level is high and excess GSH promotes the progression of the tumor, where high concentrations correlate with greater metastases [109]. When the hydrogel containing disulfide bond enters the high GSH environment, the disulfide bonds are broken, resulting in degradation of hydrogel and chemotherapy drugs delivery, such as doxorubicin, to achieve the purpose of inhibiting tumor growth. Although dynamic covalent bonds can respond to many factors, the way of stimulus response in biomaterials needs to be carefully considered. For example, dynamic covalent bonds should be sensitive enough to the temperature and pH due to a narrow range of regulation under physiological conditions, which is a remaining challenge. In addition, in the use of intelligent drug delivery system and tissue repair, different degrees of force stimulation will occur with the changes of physiological processes, and it is effective to develop drug delivery system using endogenous stress changes. On the other hand, hydrogels stimulated by exogenous and remote forces, such as magnetic field and ultrasonic pulse, could accurately release drugs under specific time and space requirements, which is also an important strategy for the development of intelligent drug delivery system. Furthermore, illumination is also a non-contact and remote controllable stimulus factor [110]. Note that the stimulation of hydrogel scaffolds in vivo requires a long-wave light source with strong penetration, while photoresponsive dynamic bonds, such as disulfide bonds and diselenide bonds, are only responsive to short-wavelength light [44,111]. Dynamic covalent bonds can also be used as sacrificial bonds to dissipate energy for polymer network and enhance the stress relaxation of materials, providing possibilities for bionic [112,113]. 

## 3. Dynamic Covalent Chemistry Endows Novel Properties of Hydrogels

Utilizing dynamic covalent bonds is an undoubted breakthrough for the hydrogel materials design. The dynamic covalent hydrogel (DCH) networks not only maintain the macroscopic mechanical stability of hydrogels, but also accelerate their microscopic dynamics, making the “liquid-like” materials. Here, we focus on the unique properties of DCH from three aspects: “liquid-like network”, in which hydrogel networks are more viscoelastic; “liquid-like interface”, makes the hydrogel interfaces better to achieve self-healing performance; and “liquid-like entirety”, which makes the hydrogel of injectable and shapeable properties, respectively.

### 3.1. “Liquid-Like Networks” Change Dynamic Mechanical Properties

**Figure 4:**
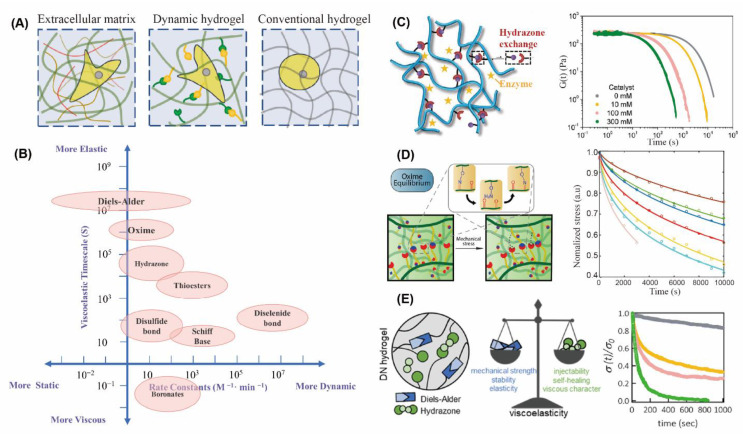
The dynamic covalent hydrogels enable highly tuned viscoelasticity (**A**) Similar to ECM, DCHs have viscoelasticity, in which cells can spread, migrate and proliferate, while permanent covalent network restricts cell behaviors; (**B**) dynamics and viscoelasticity of different DCCs. (**C**) The viscoelasticity of hydrogels was adjusted by decouple crosslinking density and exchange kinetics of crosslinks within organic catalysts [123]. Copyright © 2021 Wiley-VCH GmbH (**D**) The alginate hydrogels exhibited highly tunable stress relaxation and mechanical properties, which can be achieved by systematically varying the composition (concentration, polymer mixing ratios, degree of oxidation of NaAlg-Ald) to change crosslink density [124]. Copyright © 2019, American Chemical Society; (**E**) double network (DN) hydrogels, containing Diels-Alder adducts and hydrazone bonds, whose viscoelasticity could be highly tuned by changing the ratio between the two types of crosslinks [125].

Cell is the smallest functional unit of body. Cell research is the basis of understanding organs, tissues, and systems to explore secrets of life. At present, most of our knowledge about cell perception of external information comes from 2D cell culture. Notwithstanding this, researchers also realize that 2D cell culture is greatly different from 3D condition, and they have gradually established 3D culture platforms in vitro for cell biophysical signal perception and organoid culture [114]. However, substantial research over the past two decades developed many strategies to assess the effect of matrix stiffness on cell fates [115,116,117]. ECMs and tissues combining the properties of both solid and liquid are not linearly elastic materials—they exhibit far more complex mechanical behaviors (Figure 4A), including viscoelasticity (a time-dependent response to loading or deformation) [118,119,120]. Collagen or fibrin fibres form weak bonds in the ECM, which facilitate stress relaxation as they favor the relative displacement of fibers and energy dissipation following the application of force [121,122]. These weak bonds can present load-dependent dynamics and break, leading to the formation of new bonds and potential plastic deformations. As for the tissue, they need to exhibit time-dependent mechanical response and dissipate a fraction of the energy it took to deform them. For example, skin shear requires rapid recovery of deformation, and bone impact requires energy dissipation to avoid impact injury. Hydrogel materials need to show suited viscoelasticity similar to in vivo to be prefect candidates for ECM and tissue. As the viscoelastic response of hydrogels is determined by the rates of thermally induced dissociation of reversible crosslinks, its modulation by introduction of several types of dynamic covalent bonds with different characteristic lifetimes has recently become a hot topic. The dynamic covalent network is even more liquid-like under an imposed deformation. Unlike the elastic network, which stores all its energy internally, it can dissipate energy through dynamic covalent bonds reversible fracture and recombination.

**Figure 5:**
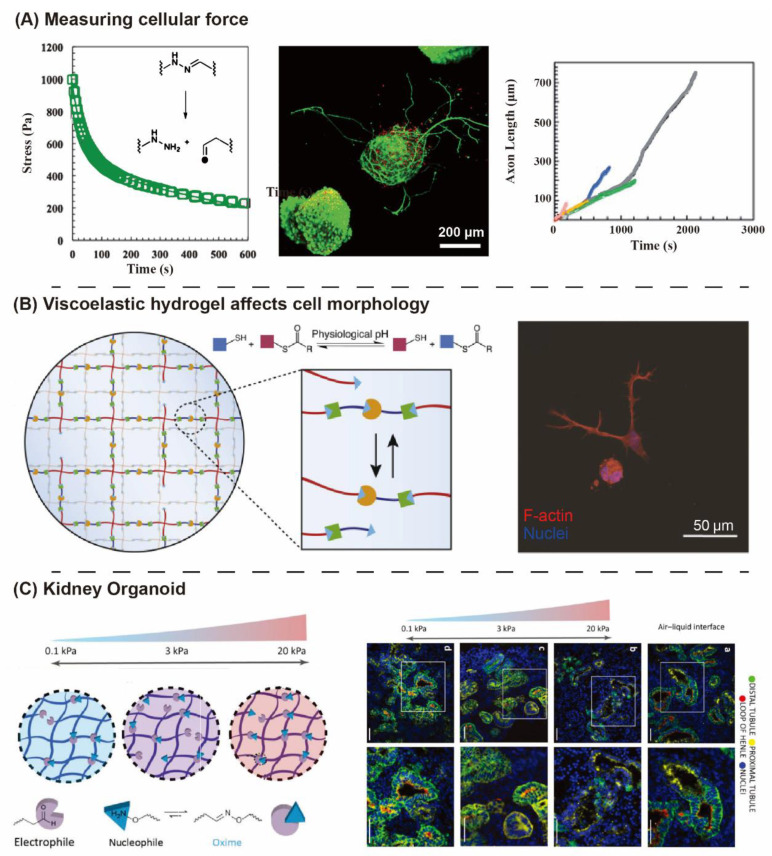
Applications of viscoelastic dynamic covalent hydrogels. (**A**) Stress relaxation of dynamic hydrazone crosslinks allows motor neurons to extend axon bodies into the hydrogels [127]. Copyright © The Royal Society of Chemistry 2014 (**B**) Thioester exchange facilitates spreading, proliferation, and migration of hMSCs in 3D scaffolds [57]. Copyright © 2018 Elsevier Ltd. (**C**) Oxime hydrogels were used to culture kidney organoids [126].

Different tissues show diverse characteristics of relaxation, and hydrogels can match different requirements of relaxation through DCC selection, external stimulation, crosslinking density, and dual network design. First, the reversible kinetic constant of DCC can affect the stress relaxation rate of hydrogels. As shown in Figure 4B, along with the increasing of rate, the DCC becomes more dynamic, the fluidity of the network increases and the relaxation is faster. Second, based on the responsiveness of dynamic covalent bonds to special external stimuli, the stress relaxation can be adjusted under some physical, chemical, and biological stimuli. Lou and coworkers developed a new strategy to decouple crosslinking density and exchange kinetics of crosslinks in viscoelastic hydrogels by using an organic catalyst (Figure 4C). Varying catalyst concentrations allowed for tuning of the exchange kinetics of dynamic covalent hydrazone crosslinks over two orders of magnitude without affecting the equilibrium constant, which provided a convenient means to control the stress relaxation behavior of viscoelastic hydrogels [123]. Viscoelasticity can also be changed by adjusting the crosslinking density of hydrogel network (Figure 4D), Sánchez-Morán developed a new synthesis route to introduce alkoxyamine functional groups into the alginate polymer backbone (NaAlg-AA) and mixed with aldehyde-containing oxidized alginate (NaAlg-Ald) to form oxime crosslinked alginate hydrogels [124]. They have demonstrated that highly tunable stress relaxation and mechanical properties can be achieved by systematically varying the composition (concentration, polymer mixing ratios, degree of oxidation of NaAlg-Ald) to change crosslink density. In addition, a dual-network hydrogel containing different-lifetime crosslinkers was designed to tune the viscoelasticity and the dynamic character of hydrogel. Mihajlovic and coworkers have investigated the fabrication of chondroitin sulfate/hyaluronic acid (CS/HA)-based DN hydrogels containing Diels–Alder adducts and hydrazone bonds, whereas the viscoelasticity could be directly tuned by changing the ratio between the two types of crosslinkers (Figure 4E) [125]. Viscoelastic hydrogels can be used to study the influence of matrix relaxation on cell morphology (Figure 5B), migration and proliferation, as well as to understand the cell–cell and cell–ECM interactions (Figure 5A). In addition, the hydrogels similar to tissues can be used for organoid incubation (Figure 5C) [57,126,127,128].

### 3.2. “Liquid-Like Interfaces” Outcome Self-Healing Properties

**Figure 6:**
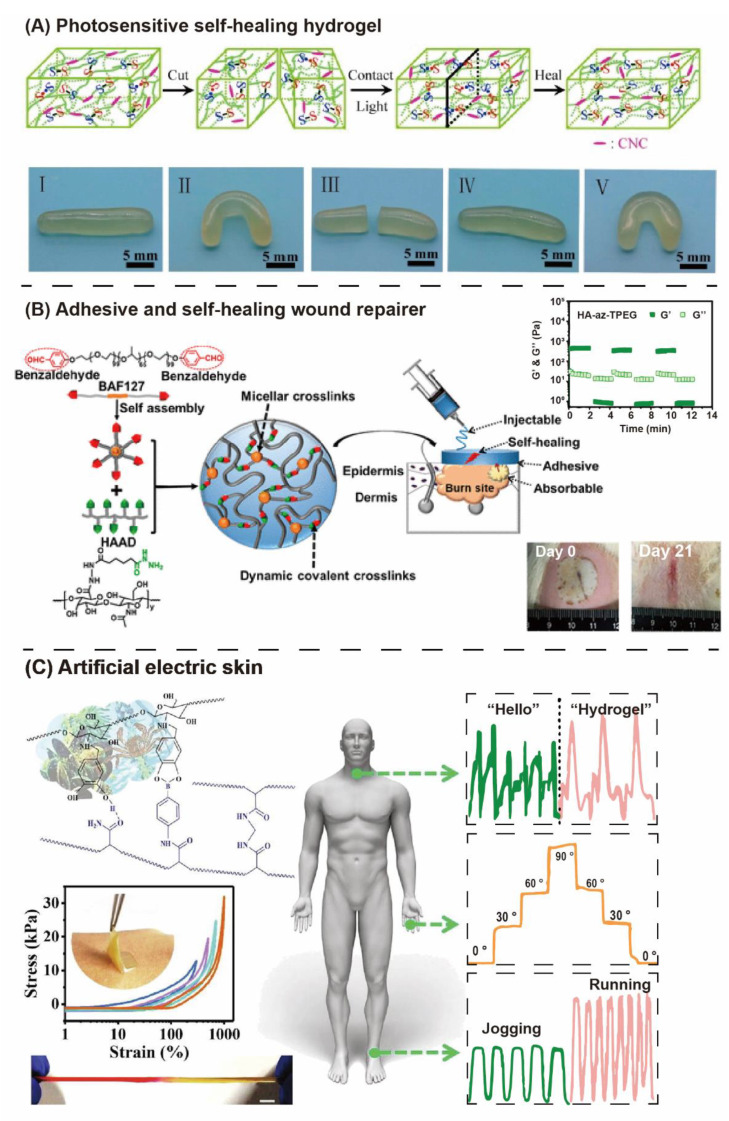
Applications of functional self-healing dynamic covalent hydrogels. (**A**) cellulose-based self-healing hydrogel based on disulfide bond photoresponse [131]. (**B**) Ha-AZ-F127 hydrogel with dynamic covalent chemically and micellar physically double-crosslinked networks was applied in the deep partial-thickness burn model to promote burn wound repair [133] Copyright © 2018, American Chemical Society. (**C**) Biocompatible ionic gels with shape-adaptability and skin adherence were used as artificial electric skin [138]. Copyright © 2020 Elsevier B.V.

Compared with other polymer materials, hydrogel materials have low mechanical properties and are prone to cracking. As these cracks are further extended, the structure of the hydrogel network is destroyed, its mechanical properties are significantly reduced, and its original function is lost, resulting in a waste of resources. Self-healing properties will increase the service life of the material, so many self-healing hydrogels have been developed [129]. Covalent Adaptive Network (CAN) hydrogels have dynamic covalent bonds at the fracture interface, and the liquid-like hydrogels have more easily interfacing bonding than the solid-like hydrogels. By recontacting the fracture surface, the polymer chain segments interpenetrate and reestablish the dynamic crosslinking sites in the damaged area to repair the network structure of the hydrogel and restore its original morphology and mechanical properties [130]. Generally, the dynamic constants of dynamic covalent bonds affect the rate and degree of self-healing of materials; meanwhile, light, pH, and temperature irritants also can regulate the healing rate. For example, Li reported a photosensitive cellulose-based self-healing hydrogel (Figure 6A) [131]. Using the photoresponsiveness of disulfide bond, the hydrogel can self-heal quickly in two minutes. In addition, the higher the hardness of hydrogel, the worse the self-healing effect of the material. Of course, self-healing ability is not only simple repair morphology and mechanical properties, but more importantly, it endows hydrogels with novel properties, such as adhesion-based DCC. Owing to their ability to self-heal, dynamic covalent hydrogels are intrinsically adhesive and possess high cohesion on account of the strong covalent bonds that hold the networks together [132]. Adhesives and hydrogel interfaces include chemical properties for reversible adhesion, which are formed with complementary functional groups. Adhesion can be easily relieved after stimulants. This hydrogel can be used as wound sealer and repair. For example, Li and coworkers prepared Ha-AZ-F127 hydrogel (Figure 6B), with DCC and micellar physically double-crosslinked networks exhibited rapid gelation and shear thinning properties [133]. Afterwards, it was applied in the deep partial-thickness burn model, and the hydrogel contributed effectively in promoting burn wound repair. DCH with adhesion and self-healing, or the addition of a conductive segment (such as polypyrrole and graphene) to increase conductivity, could be used as a flexible electronic device or artificial electric skin, which is capable of strongly adhering to different parts of human bodies and precisely detecting different types of human motions, showing great promise for biomedical prosthetics, human/machine interfaces, wearable devices, and soft robotics [134,135,136,137]. Wang and coworkers have designed biocompatible ionic gels with shape-adaptability and skin adhesion, which contain reversible crosslinkers of H-bonding and dynamic covalent bonds (Figure 6C) [138]. The ionic gel skin has strong adhering strength, rapid self-healing in minutes and large stretchability, furthermore, which is capable of directly cutting to human skin to sense both subtle and drastic human motions without showing any detaching.

### 3.3. “Liquid-Like Entireties” Turn out Injectable and Shape-Shifting/Memory Materials

**Figure 7:**
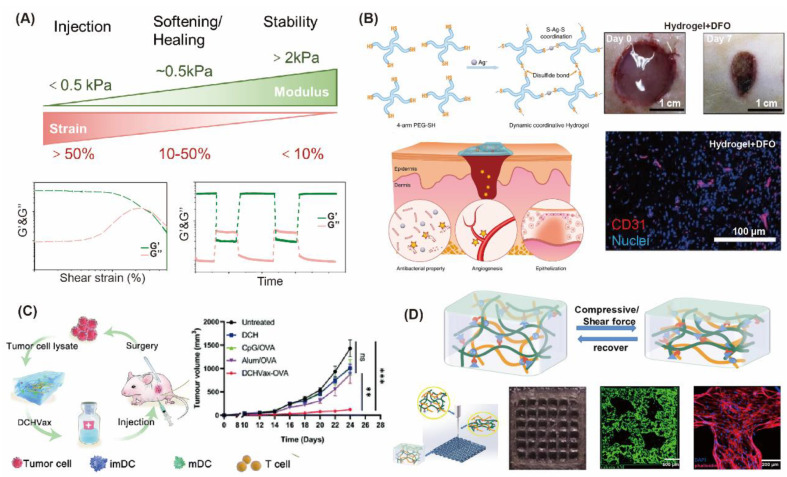
Applications of injectable dynamic covalent hydrogels. (**A**) typical rheological behaviors of injectable hydrogels. (**B**) Hydrogel containing dynamic nature of Ag-S coordination bond has injectable and self-healing properties. After loading DFO, hydrogels can realize angiogenic properties for diabetic wound regeneration [86]. (**C**) Subcutaneous injection of dynamic covalent hydrogel-based vaccine (DCHVax) recruits dendritic cells to the matrix in situ and elicits robust tumor-specific immune responses [142]. (ns: not significant, ** *p* < 0.01, *** *p* < 0.001) (**D**) A double-network (DN) hydrogel based on dynamic hydrazone-crosslinked hyaluronic acid (HA-HYD) photocrosslinked gelatin methacrylate (GelMA), which was suitable for extrusion-based 3D printing, in which BMSCs could proliferate well [144].

The liquid-like properties of DCH are not only shown in the network and interface, but also in its entirety of injectable, shapeable, and shape memory characters. Injectable hydrogels become increasingly important in the fields of tissue engineering and drug delivery due to their tunable properties, controllable degradation, high water content friendly to drugs and cells, and the ability to deliver them in a minimally invasive manner [139]. As hydrogels are injected into the body, they meet the need for customization and maintain gel volume and shape for a certain period of time to aid tissue repair, drug delivery, and cell therapy. For injectable materials, liquid-like and solid-like relationships need to be balanced to resolve the conflicts between injectability and stability. Conventional permanent crosslinked hydrogels are non-flowable because covalent bonds are too stable to rupture and prevent the intermolecular relative movement. However, DCH dissipate stress by reversible fracture of DCC under deformation or high shear, resulting in liquid-like behavior. After stress removal, DCC reproduces network self-healing and retains solid-like properties [140]. From the typical rheological curves of injectable hydrogels (Figure 7A), they usually undergo a transition from a static equilibrium state to a nonlinear elastic state and finally back to a static equilibrium state. In traditional treatment methods, drugs need to enter the body through intravenous or oral administration, and then it takes a certain time for the drugs to reach the lesion and exert a curative effect. Hydrogel loading drugs or cells can be injected, and disease treatment can be carried out through minimally invasive way, which shows the ability of fast effects, long duration and reduces the pain of patients. Chen et al. have reported that a hydrogel containing dynamic nature of Ag-S coordination bond possesses injectable and self-healing properties [86]. Due to the antibacterial properties of Ag^+^, hydrogel is antibacterial. Synchronously loading an angiogenic drug in the system, desferrioxamine (DFO) can realize angiogenic properties for diabetic wound regeneration (Figure 7B). According to their another report, adhesive liposomes (A-lip) loaded with BMP-2 enhanced the in vivo adhesion of Ag-S coordination hydrogels and improved osteogenic differentiation and faster local bone remodeling of osteoporotic fractures in rats [141]. Chen and coworkers have reported a dynamic covalent hydrogel based vaccine (DCHVax), which used proteins extracted from the resected tumor as antigens, CpG as the adjuvant, and a multi-armed poly(ethylene glycol) (8-arm PEG)/oxidized dextran (ODEX) dynamically crosslinked hydrogel as the matrix. Subcutaneous injection of DCHVax recruits dendritic cells to the matrix in situ and elicits robust tumor-specific immune responses (Figure 7C) [142]. This simple and personalized method to develop cancer vaccines may be promising in developing clinically relevant strategies for postoperative cancer treatment. Liu’s group develops Schiff-based hydrogel encapsulation of functionalized MSC aggregates (FMAs) for treating myocardial infarction. DCH embedded stem cells will be widely studied and applied in the field of cell therapy and tissue repair [143]. Injectable hydrogels can also be used as bioinks to print tissue engineering. Wang reported a double-network (DN) hydrogel based on dynamic hydrazone-crosslinked hyaluronic acid (HA-HYD) photocrosslinked gelatin methacrylate (GelMA), which was suitable for extrusion-based 3D printing [144]. The 3D scaffolds with uniform filaments and pore size were printed layer by layer, and subsequent photocrosslinking increased the mechanical strength, together with the self-healing of the DN hydrogel that created a scaffold with an integrated and stable structure. The proliferation of BMSCs in the printed scaffold was obvious, and the modified scaffold could be developed as tissue engineering repair scaffold (Figure 7D).

**Figure 8:**
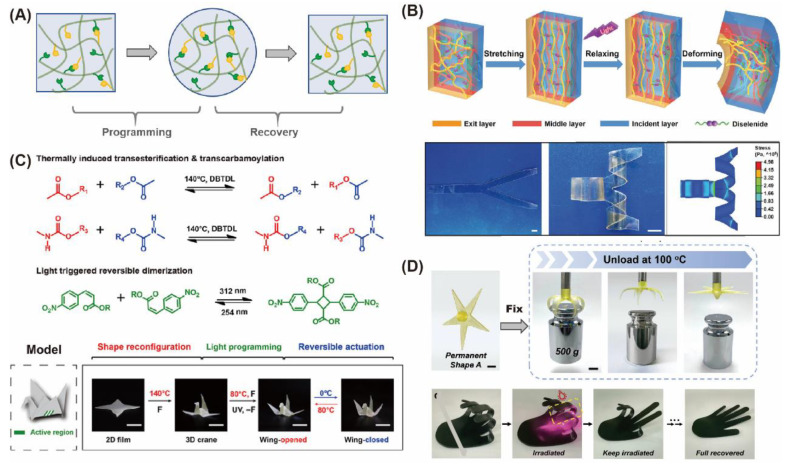
Shape memory dynamic covalent hydrogels. (**A**) shape memory process of dynamic covalent bond hydrogels; (**B**) shape programming through the light-induced dynamic diselenide exchange for tensile stress relaxation [149]. Copyright © 2021 Wiley-VCH GmbH; (**C**) thermally induced transesterification and transcarbamoylation and photo-reversible dimerization of nitro-cinnamate. Spatioselective reversible actuation in complex 3D objects [150]. Copyright © 2022 Wiley-VCH GmbH; (**D**) 4D printing polythiourethane (4DP-PTU) from dynamic thiocarbamate could be self-healing and remodeling [151]. Copyright © 2022 Wiley-VCH GmbH.

Shape memory is one of the unique properties of smart materials. The shape memory process must undergo a deformation-formation-recovery process (Figure 8A). Upon an external force, the fluidity of molecular chain increases, and molecular interaction changes from weak to strong or from absence to existence, forming a “lock” fixed deformation. Under another stimulus, the temporarily stronger interaction is broken, and the chain rearranges again and returns to original shape [145,146]. A covalently crosslinked network can only form permanent shapes and is difficult to reshape, which limits the complexity and diversity of material deformation. 4D printing is an advanced version of 3D printing. The process of 4D printing is when a printed 3D object becomes another structure due to the influence of outside energy inputs such as temperature, light, or other environmental stimuli. The introduction of dynamic covalent bonds into shape memory materials can lead to the development of 4D printing smart materials with high-strength, multi-reconfigurable, self-healing and recyclable, and be used for application in intelligent devices, biomedicine and tissue engineering [147,148]. Xu and Liu reported shape-memory polymers (SMPs) using a diselenide dynamic covalent bond to turn the material from 2D sheets to 3D configurations through light programming. The materials can maintain and release internal stress through shape memory effect, thus simplifying the programming setup (Figure 8B) [149]. Xie et al. devised a strategy that used a programmable crystalline shape memory polymer with thermo- and photo-reversible bonds to create a single-component robot. This shape memory material has a wide temperature difference window in shape programming and recovery regulation, and resulted in reversible actuation of complex 3D structures (Figure 8C) [150]. Zhang and coworkers obtained 4D printing polythiourethane (4DP-PTU) from dynamic thiocarbamate bonds. The polymer material is self-healing and remodeling, and the 4DP-PTU doped with carbon nanotubes enables precise localized shape control triggered by near-infrared light (NIR) for 4D printing. Meanwhile, the material has high biocompatibility and changes cell adhesion by surface modification on demand, which is of great significance for the performance of 4D printed biological implants in vivo (Figure 8D) [151].

## 4. Conclusions and Perspectives

Owing to DCC, an increasing number of soft hydrogel materials have turned into adaptive/dynamic or smart hydrogels from static hydrogel. Gelation by using dynamic covalent crosslinking endows the hydrogel with excellent properties to meet the demands in biomedicine and biotechnology applications. In this review, we highlight the conventional DCC derived from different chemical reaction mechanisms. Additionally, we provide details on the mechanical strength of these dynamic covalent bonds and their kinetics. The mechanical force of DCC varies greatly with different dynamics, just like mechanical strength of disulfide and diselenide are strong enough over 1 nN, while thioester and coordinate bond are about several hundred pN. By introducing catalyst into the solution, the rupture forces of S-S decrease to ~100 pN, which can be easily dissociated by external mechanical stimuli. However, they are still much stronger than that of non-covalent interactions which can maintain relative high strength. Then, we illustrate the applications of dynamic covalent hydrogels network hydrogels: (1) DCC changes the network structure and makes the hydrogel more viscoelastic, contributing to the development of biomimetic ECM materials. (2) The DCC increases interfacial fluidity of hydrogel, resulting in self-healing properties, broadening the hydrogel applications in hemostatic adhesive, drug carries, adhesive hydrogel preparations, and flexible conductive material functional self-healing materials. (3) They also possess overall liquidity performance, and exhibit injectability and shape memory, which is excellent for applications in the field of medicine and intelligent materials. 

Although DCC has made great progress in soft hydrogel materials, such as self-healing, injectable and suitable for post-processing and additive manufacturing, many challenges in synthetic and design aspects remain to be discovered. For example, how can we predict and rationally design a hydrogel with proper mechanical properties? The kinetics of the dynamic covalent bonds must be known. However, it is hard to obtain the kinetics in single molecule level. Although the technology of single molecule force spectroscopy provides a great way to characterize the molecular kinetics, new methods of measurement are urgent. Another challenge relies on the design of hydrogels with a spatial inhomogeneity in mechanical properties that local material properties cannot be represented by bulk properties that are suitable for cell culture. Measuring the local mechanics and studying how cells respond to local biophysical cues are challenging. Ultimately, dynamic covalent chemistry greatly promotes the development of soft hydrogel materials and broadens their applications in biomedicines and biotechnologies.
